# Cancer Stage, Comorbidity, and Socioeconomic Differences in the Effect of Cancer on Labour Market Participation: A Danish Register-Based Follow-Up Study

**DOI:** 10.1371/journal.pone.0128621

**Published:** 2015-06-01

**Authors:** Karsten Thielen, Christophe Kolodziejczyk, Ingelise Andersen, Eskil Heinesen, Finn Diderichsen

**Affiliations:** 1 Section of Social Medicine, Institute of Public Health, University of Copenhagen, Copenhagen K, Denmark; 2 KORA, Danish Institute for Local and Regional Government Research, Copenhagen K, Denmark; 3 Rockwool Foundation Research Unit, Copenhagen K, Denmark; Academic Medical Center, NETHERLANDS

## Abstract

**Purpose:**

Socioeconomic inequality in return to work after cancer treatment and rehabilitation have been documented, but less is known about its causes. This paper investigates the role played by breast cancer stage at diagnosis and comorbidity.

**Methods:**

We used the comprehensive Danish Cancer Registry to follow 7372 women aged 30-60, who were in the labour force when diagnosed with breast cancer in 2000-06 and survived at least three years. Controls were 213,276 women without breast cancer. Inequalities in employment outlook were estimated as interaction effects in linear regression between educational attainment and disease on employment.

**Results:**

There is significant interaction between education and breast cancer, but it is only marginally affected by including stage and comorbidity in the regression models. Education, breast cancer stage, and comorbidity all have strong effects on later employment, and a considerable amount of the educational effect is mediated by comorbidity and pre-cancer labour market participation and income.

**Conclusion:**

The result of the study is negative in the sense that the stronger effect of breast cancer on employment among low-educated compared to highly educated individuals is not explained by cancer stage or comorbidity. The fact that comorbidity has little impact on inequality may be due to a different social patterning of most comorbidity compared to breast cancer.

## Introduction

Social inequality in burden of disease is generated not only by inequality in incidence but also by inequality in the consequences of disease such as survival, disability, and labour market participation.[1] For several cancer locations, the negative effect of lower socioeconomic position (SEP) is, for example, much more pronounced for survival than for incidence.[2] One indicator of inequality in disability and labour market participation is the large educational difference in employment rates among those with limiting long-term illness in general [3] and cancer patients in particular.[4] Differential employment consequences for cancer patients might occur through several mechanisms: a) Differences in taking part in screening programmes [5] and in awareness of signs and symptoms might delay diagnosis and make treatment less effective;[6] and b) inequality in co-morbidity and exposure to behavioural and environmental factors might influence course of disease;[7] c) inequality in quality and effectiveness of treatment and rehabilitation;[8] d) different labour market conditions, including employers’ willingness to retain employees or hire people affected by illness, and flexibility in adjusting working demands and supporting conditions. Denmark is characterised by low employment protection for sick employees but high economic security and high spending on active labour market policy.[3] This paper deals with the first two of the above mechanisms.

We have focused on breast cancer since it leaves less severe functional limitations compared to most other cancer types following successful treatment and since there is no negative effect of educational attainment on incidence—in fact, the opposite is true.[2] Breast cancer survival has improved steadily. 5-year survival in Denmark was 74.7% in the period of 2007–09, an improvement of 18% compared to 1998–2000.[9] Due to a growing incidence and improved survival, the prevalence of breast cancer survivors is increasing steadily by approximately 2% per year.[10] 5-year survival is 8% higher among high educated individuals.[2]

Several factors influence the probability of employment after diagnosis and treatment. Premorbid employment status, education, income, and extent of malignancy (local, regional, or distal spread) as well as type of treatment have been shown to influence employment status in the years following diagnosis and treatment.[11,12] A Danish study showed a strong influence from low income and co-morbidity on the risk of disability pension among cancer survivors.[13] A recent Norwegian study also showed interactions indicative that the effect of educational attainment on employment was stronger among cancer survivors than among healthy controls while the effect of income was, in contrast, stronger among controls.[12] One potential explanation behind the unequal social consequences of cancer might be that people with lower SEP are diagnosed at a later stage.[14] In Denmark, where there is no co-payment for referral to specialist treatment, it has been shown that delayed diagnosis might be due to both patients’ and doctors’ behaviours and attitudes.[15]

Based on the findings of a clear educational gradient in employment consequences of cancer, [4] this paper will identify the role played by stage of disease at time of diagnosis and comorbidity in explaining the social gradient in later employment status. This prospective study uses as its empirical basis a Danish population of women aged 30–60 to analyse the following objectives:

Estimate the extent to which educational differences in breast cancer stages at diagnosis explain educational differences in labour force participation and disability pension three years after diagnosis.

Estimate the extent to which the effect of cancer stages is modified by educational differences in comorbidity.

## Material and Methods

### Cases and controls

**Fig 1:**
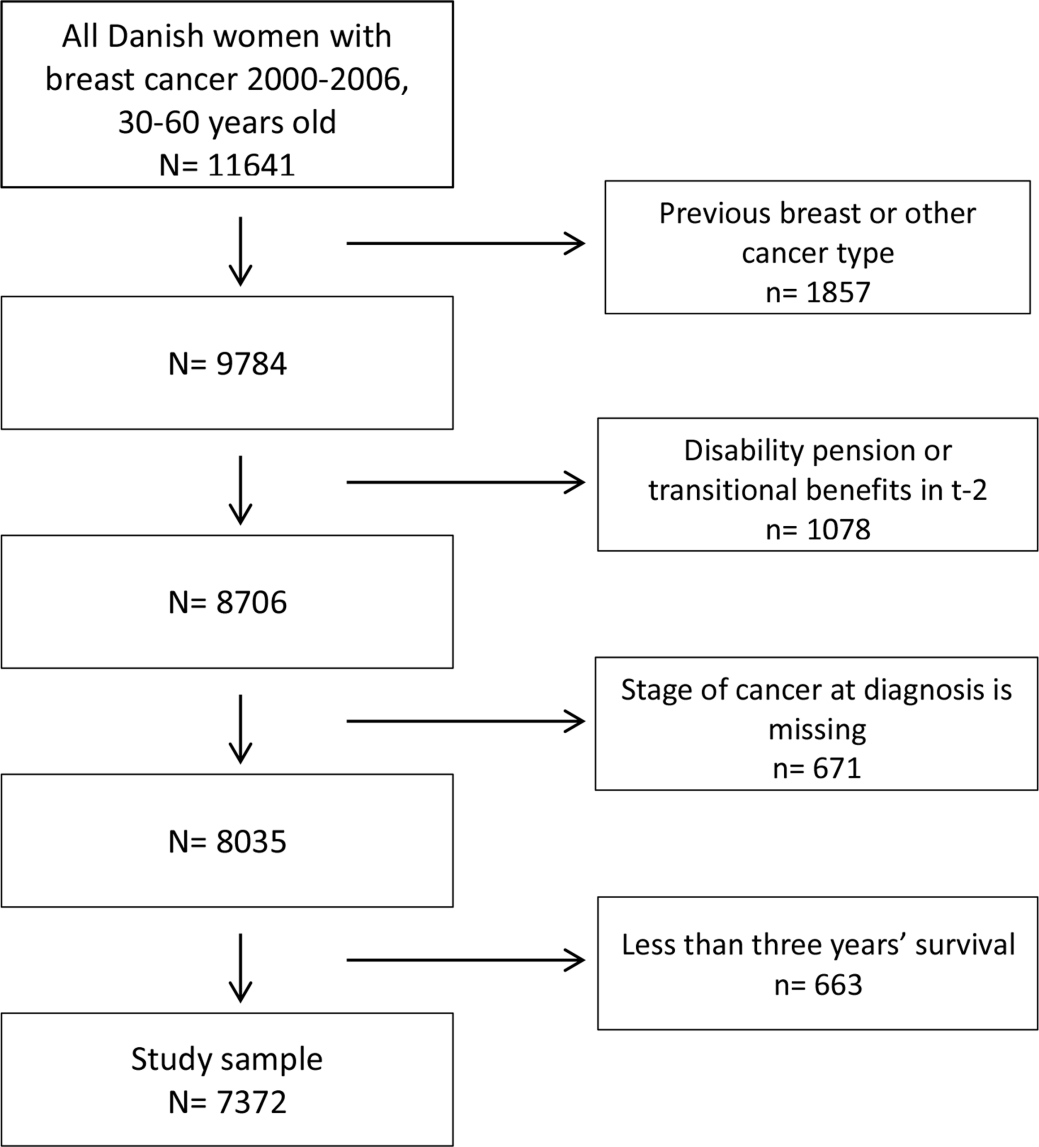
Study population, exclusion process, and final study sample size.

Social inequalities in consequences of disease are often studied among cases only. This approach provides a relevant but purely descriptive result. If, in contrast, we wish to estimate the effects of disease on employment and the extent to which the effect is modified by education, stage at diagnosis, etc., we must include healthy controls in the study and estimate interaction between disease, stage, and education. We have therefore designed a register-based prospective study. We identified all females in the Danish Cancer Registry who received a diagnosis of breast cancer (ICD10 code C50) in the period of 2000–2006 (base years). From all 28,367 registered cases of breast cancer in Denmark in 2000 to 2006, a total 7372 cases were included in the study, fulfilling the following criteria: they (1) were living in Denmark; (2) had no cancer registered prior to the breast cancer incident case; (3) were aged 30 to 60 in the year of diagnosis; (4) survived at least to the end of the third year following diagnosis; and (5) received no disability pension or so-called transitional benefits in the two years preceding the year of diagnosis (since those with transitional benefits can be regarded as already on their way out of the labour market to early retirement). This means that all women who were part of the labour force, including those on temporary benefits due to sickness or unemployment, were included (Fig 1).

Controls were collected from the population register covering all citizens of Denmark. For each of the base years 2000–2006, the controls consist of females living in Denmark, aged 30 to 60, who survived at least three years after the base year and who had no cancer diagnosis before or after the base year. For the analysis for each year, we selected a random sample of 4% of women meeting the aforementioned criteria with a total of 213,276 controls. The procedure for randomly selecting the control group ensures that no person is in the control group for two different base years.

The Danish civil registration number (CPR), which is a ten digit PIN-code unique to every Danish citizen, makes it possible to link cancer registries with other official registry data on demographics, education, employment, occupation, income, municipality of residence, contacts to general practitioners, hospitalisations, and purchase of prescription drugs.

### Exposure

Data on cancer was obtained from the Danish Cancer Registry, which is a population-based registry containing data on all incident cases of cancer throughout Denmark since 1943. The Cancer Registry contains information related to tumour characteristics, including tumour stage.[16] For this investigation, we categorised breast cancer patients into one group with cancer at a *localised* stage (not yet entered the lymphatic system) (covering 3,769 women) and another group of 3,603 women with regional lymphatic spreading or any other kind of *metastasised* cancer.

SEP was defined by educational attainment. Information on education was obtained from registries for the baseline year. Education was classified into three categories, in accordance with the International Standard Classification of Education (ISCED) system (UNESCO 1997): *Compulsory* (up to 10 years of education, ISCED Levels 0–2), *Vocational* (up to 11–12 years of education, ISCED Levels 3), and *Further* (13 or more years of education, ISCED Levels 4–6).

### Confounders

Adjustments for a large number of covariates in the analyses were made with regression in a linear probability model. A linear model was applied as we find absolute effects more relevant for policy than relative effects. Covariates were chosen among factors that are known to be associated with both labour market outcomes and cancer.[15] *Age* was included in the analyses as a continuous variable covering the ages of 30 to 60. Information on *family type* was categorised into three groups: single with children, married with or without children, and cohabiting with or without children. Information on *ethnicity* was dichotomised as Danish born yes/no. Since prognosis on cancer could be affected by *access* to and *quality* of the *regional* health service system, we controlled for the five regions in Denmark (using four dummy variables) as well as size and degree of urbanisation of the municipality in which the study participants lived (using 11 dummy variables).

*Premorbid labour market status* was carefully controlled for: Persons on disability pension and transitional benefits two years before diagnosis were excluded, but the very few homemakers and those on more temporary benefits such as sickness or unemployment benefits were included as they might return to work. Labour market status two years before diagnosis as well as change in unemployment, self-employment, occupation, industry, and part-time employment status from two to five years before diagnosis were, however, controlled for in detail. We furthermore measured individual income two years before diagnosis. *Comorbidity* was also measured and controlled for by several items: Number of contacts with GP, hospitalisations (dummy variables for the 18 ICD-10 chapters (infectious disease or diseases of blood, etc.; endocrine, nutritional and metabolic; mental; nervous system; eye and adnexa; ear and mastoid process; circulatory system; respiratory system; digestive system; skin and subcutaneous tissue; musculoskeletal system and connective tissue; genitourinary system; pregnancy, childbirth and the puerperium; congenital malformations, deformations and chromosomal abnormalities; ill-defined conditions; injury, poisoning, etc.); contacts with health services and factors influencing health status; and medicinal prescriptions (dummy variables for the indications: blood pressure; heart disease; rheumatism; hormonal preparation; diabetes; antipsychotic; antidepressant, analgesic; hypertension; Parkinson; osteoporosis; asthma; bronchitis; thyroid; anti-thyroid; antiepileptics; anxiolytics; acid disorders; acid anti migraine; headaches). We decided not to use an established comorbidity index like the Charlson index because none of these are developed to predict employment consequences. We controlled for comorbidity variables measured at two to five years before the base year since health information measured in the base year or the year before may be affected by the cancer, especially if it is diagnosed at a late stage.

### Endpoints

The study’s endpoints are measured from registry-based information. To be registered with a certain employment status, this status must be the dominant status for the year. Labour market affiliation three years after diagnosis was categorised for those who permanently or temporarily *left the labour force*, including those on disability pension, sickness benefits, early retirement pension, social assistance, and supported by a spouse. We have also looked specifically at *disability pension*, which is given to people with medically certified *permanently* reduced working capacity and has important public health implications since it rules out return to work for the individual and results in long-term societal costs.

### Statistical methods

We use a linear probability model to model the effect of cancer stage as a predictor of being out of the labour force or of receiving a disability pension. This involves running a regression of the two outcomes on a series of covariates, including dummy variables for cancer stage by ordinary least squares. As the outcomes are dichotomous variables, the estimates of the model are directly interpreted as the effect of the corresponding covariate on the probability that the outcome is equal to 1. The model has been estimated using the ‘Regress’ command from the statistical software package Stata 13.1. Furthermore, standard errors are corrected for in terms of the presence of unknown form heteroskedasticity, using the method suggested by Davidson and MacKinnon.[17] We estimated the joint effect of education and cancer stage by including in the model interaction terms between education levels and cancer stage. A dichotomous outcome often leads to the choice of a logistic regression model. However, in this case we consider an additive linear probability model as more appropriate, because we are interested in marginal effects of cancer, education and their interaction. Furthermore, in contrast to the multiplicative logistic model, the coefficients of a linear probability model may be interpreted directly in terms of absolute percentage point differences, which we think is more public health relevant and more intuitive to interpret and communicate. Finally, even though the true model is non-linear, it is not obvious that, e.g., the logistic model would be the correct specification.

For each of the outcomes, we present the resulting estimates from three different models. Model 1 is adjusted for age, year of diagnosis, family type, ethnicity, region, and education. In this model, disease stage is not taken into account, i.e. the stage dummies are replaced by a single dummy variable for whether the individual has had cancer (denoted *Cancer*). In Model 2, we further adjust for the stage of cancer as well as all interactions between stage and education. In Model 3, we further adjust for comorbidity defined by 18 indicators for hospitalisation for specific diagnoses, 20 indicators for consumption of prescribed drugs, number of GP contacts, and for premorbid labour market status and income.

### Ethics statement

The study was approved by the Danish Data Protection Agency. The registry data was analysed at Statistics Denmark. At Statistics Denmark, the original CPR numbers are replaced by other personal identification numbers in order to make the people in the dataset anonymous to the researchers. Consequently, ethical approvals are unnecessary.

## Results

Table 1 shows that the distribution of cancer stage at diagnosis is very similar for the three education groups. Two years before the base year, women with compulsory education had, as expected, the highest rate of co-morbidity, and the percentage temporarily not employed (i.e. sick or unemployed) was twice as large for this group as for those with longer educations. The percentage of women living in the capital region was considerably higher among the group with a further education. For the outcomes three years after the base year, the share of people out of the labour force and the share receiving disability pension were about three times higher for those with only compulsory education than for the group with a further education.

**Table 1:** Prevalence of some major covariates and outcomes by educational level.

	Compulsory Education N = 55,334	Vocational Education N = 82,832	Further Education N = 82,482
*Covariates*			
% localised cancer	1.8	1.7	1.7
% metastasised cancer	1.7	1.6	1.6
% with any comorbidity	73.2	66.8	59.1
% temporarily not employed t-2	23.1	10.4	10.2
% in Capital Region	25.8	26.3	36.7
*Outcomes*			
% out of labour force	26.0	12.1	8.5
% on disability pension	6.6	2.6	1.8

N = sample size

Table 2 shows estimated results for the three linear probability models for the risk of being out of the labour force three years after diagnosis. In Model 1, the average effect of cancer on the risk of being out of the labour force for women with only compulsory education is 7.8%. The effect for women with vocational education is approximately the same since the interaction term between cancer and vocational education is negative but statistically insignificant. However, the effect for women with a further education is significantly smaller, namely 5.2% (due to a significantly negative interaction term between cancer and further education of -0.026).

**Table 2:** Effect on risk of being out of the labour force three years after diagnosis.

	Model 1		Model 2		Model 3	
	b	(95% CI)	b	(95% CI)	b	(95% CI)
Cancer vs. healthy	**0.078**	**[0.058,0.098]**				
Localised vs. healthy			**0.055**	**[0.027,0.082]**	**0.067**	**[0.043,0.091]**
Metastasised vs. healthy			**0.102**	**[0.073,0.130]**	**0.111**	**[0.084,0.137]**
Vocational vs. Compulsory education	**-0.113**	**[-0.117,-0.109]**	**-0.113**	**[-0.117,-0.109]**	**-0.042**	**[-0.046,-0.039]**
Further vs. Compulsory education	**-0.142**	**[-0.146,-0.138]**	**-0.142**	**[-0.146,-0.138]**	**-0.069**	**[-0.073,-0.065]**
Vocational education*Cancer	-0.003	[-0.027,0.022]				
Further education*Cancer	**-0.026**	**[-0.050,-0.002]**				
Vocational education*Localised			0.004	[-0.030,0.038]	-0.022	[-0.052,0.009]
Vocational education*Metastasised			-0.009	[-0.044,0.027]	-0.031	[-0.064,0.002]
Further education*Localised			-0.018	[-0.050,0.014]	-0.029	[-0.058,-0.000]
Further education*Metastasised			**-0.035**	**[-0.070,-0.001]**	**-0.044**	**[-0.076,-0.013]**
Age dummy variables	Yes		Yes		Yes	
Calendar year dummy variables	Yes		Yes		Yes	
Premorbid employment/income	No		No		Yes	
Comorbidity indicators	No		No		Yes	
Other controls^a^	Yes		Yes		Yes	
N	220,648		220,648		220,648	
Adj. R^2	0.184		0.184		0.340	

Percentage units calculated using linear probability model (OLS).

Estimates that are statistically significant at the 95% confidence level are shown in bold.

^a^ Other controls: family type, ethnicity, municipality type, and region

b = percentage unit; 95% CI = 95% confidence interval; N = sample size; Adj. R^2 = Adjusted R squared;

* = interaction of two terms

Model 2 includes the same list of aforementioned covariates as in Model 1, but the cancer dummy is replaced by a dummy for localised cancer and one for metastasised cancer. For women with only compulsory education, localised and metastasised cancer increase the risk of leaving the labour force by 5.5% and 10.2% respectively. Again, the estimated effects for women with a vocational education are similar since the interaction terms between the cancer dummy variables and the vocational education dummy are close to zero. The estimated effect of metastasised cancer for women with a further education is approximately 6.6%, i.e. 3.5% lower than for lower educated women (since the interaction term between metastasised cancer and further education is significantly negative: -0.035). The interaction terms for education and the two stages are not smaller in Model 2 than in Model 1, indicating that stage does not explain the inequality in employment prospects. The main effects of education are shown in the table. According to the estimates in Models 1 and 2, these indicate that, for women without cancer, the risk of leaving the labour force is 11% or 14% lower if they have a vocational or further education respectively compared to having only compulsory education.

In Model 3, where we control for baseline labour market status, income, and co-morbidity, these ‘education effects’ are smaller. Interestingly, the effect of cancer stages and the interaction terms between education and cancer stages are slightly larger in Model 3 than in Model 2.

Table 3 is similar to Table 2 except that the outcome is the risk of receiving disability pension (i.e. being permanently out of the labour force due to significantly and medically certified reduced working ability) three years after diagnosis. Here, all interaction terms between cancer and education are clearly significant, but introducing stages results in little change. Including comorbidity as well yields the same but smaller negative confounding effect as were evident in Table 2.

**Table 3:** Effect on being on disability pension three years after diagnosis.

	Model 1		Model 2		Model 3	
	b	(95% CI)	b	(95% CI)	b	(95% CI)
Cancer vs. healthy	**0.087**	**[0.071,0.103]**				
Localised vs. healthy			**0.064**	**[0.043,0.085]**	**0.063**	**[0.043,0.083]**
Metastasised vs. healthy			**0.111**	**[0.087,0.136]**	**0.113**	**[0.089,0.136]**
Vocational vs. Compulsory educ.	**-0.033**	**[-0.035,-0.030]**	**-0.033**	**[-0.035,-0.030]**	**-0.012**	**[-0.014,-0.009]**
Further vs. Compulsory education	**-0.039**	**[-0.041,-0.036]**	**-0.039**	**[-0.041,-0.036]**	**-0.019**	**[-0.021,-0.016]**
Vocational education*Cancer	**-0.041**	**[-0.060,-0.022]**				
Further education*Cancer	**-0.050**	**[-0.069,-0.032]**				
Vocational education*Localised			**-0.031**	**[-0.056,-0.006]**	**-0.035**	**[-0.059,-0.012]**
Vocational education*Metastasised			**-0.052**	**[-0.080,-0.023]**	**-0.059**	**[-0.086,-0.031]**
Further education*Localised			**-0.040**	**[-0.064,-0.017]**	**-0.043**	**[-0.066,-0.020]**
Further education*Metastasised			**-0.060**	**[-0.088,-0.032]**	**-0.065**	**[-0.092,-0.038]**
Age dummy variables	Yes		Yes		Yes	
Year dummy variables	Yes		Yes		Yes	
Premorbid employment/income	No		No		Yes	
Comorbidity indicators	No		No		Yes	
Other controls ^a^	Yes		Yes		Yes	
N	220,648		220,648		220,648	
Adj. R^2	0.028		0.028		0.127	

Percentage units calculated using linear probability model (OLS).

Estimates, which are statistically significant at the 95% confidence level, are shown in bold.

^a^ Other controls: family type, ethnicity, municipality type, and region

b = percentage unit; 95% CI = 95% confidence interval; N = sample size; Adj. R^2 = Adjusted R squared;

* = interaction of two terms

## Discussion

The criteria for unequal employment consequences of breast cancer in this paper are calculated as the interaction (in terms of departure from additivity) between education and disease instead of a mere description of different employment prospects across patients with different levels of educational attainment. The significant negative interaction effect of educational attainment and breast cancer on the probability of being out of the labour force following treatment is consistent with social inequality found in other studies from Scandinavia, Korea, and Japan.[18–21] The aim of this study was to assess the role of cancer stage and comorbidity as possible mechanisms. Cancer stage at time of diagnosis is important for future employment, but the fact that the strength of the interaction with educational attainment does not substantially change with the inclusion of cancer stage at diagnosis illustrates that educational differences in diagnostic delay are not an important explanation for the unequal employment prospects. To the best of our knowledge, no other study has investigated the interaction between education and breast cancer stage. As assumed, comorbidity is important for chances of returning to work, as also shown in one Danish and one Korean study,[2,18] but since the social gradient in most other types of morbidity has the opposite direction than breast cancer, the effect of cancer on employment, particularly among individuals with low educational levels, is stronger after controlling for comorbidity. The effect of low education on employment among non-cancer patients is, however, strongly mediated by comorbidity.

It might be surprising that the effect of breast cancer on disability pension is not stronger than on being out of work in general. It illustrates the fact that tiredness and other symptoms following breast cancer might not qualify a person for a disability pension but might still force many patients out of work.

Lower education increases the probability of being out of paid work in general, but the effect is stronger among women with breast cancer and stronger for those diagnosed with more advanced stages of the disease. This is potentially indicative of an unfair inequity. Returning to the two other proposed mechanisms, people with low levels of education often have to return to more physically demanding jobs following illness. As a result, the higher risk of disability pension might be well motivated by lower working ability in relation to one’s previous job.[22] However, it might also reflect inequities in the effectiveness and implementation of rehabilitation measures, as is supported by a recent Danish study, showing social inequality in participation in cancer rehabilitation.[8] The fact that disease stage does not play an important role in the effect of education indicates, however, that factors generating delay in the diagnostic process are not a major cause of inequality in employment prospects. Future research might therefore focus on the rehabilitation and return to work process in order to find explanations for inequality in employment consequences. Differences in access to, attendance at, and effect of rehabilitation measures might be important. Socially differentially patterned workplace or labour market factors may unequally increase the negative effect of functional impairment.

We excluded women who had permanently left the labour force prior to diagnosis but included those who were temporarily out of work and adjusted the analysis for these types of labour market status before diagnosis. A ‘cleaner’ estimation of the risk of leaving the labour force could have been achieved by excluding these factors.[2,22] The confounding effect of comorbidity and labour market status before cancer diagnosis does not significantly influence the labour market participation effect of cancer but tends to strengthen the modifying effect of education (Model 3). Using registry data on hospitalisations, prescriptions, and GP contacts to control for comorbidity might, however, leave some residual confounding since much morbidity might not be visible in these registries. This bias could be stronger among patients with low socioeconomic status as they might have a larger amount of unmet needs even in the Danish healthcare system.[23] But it cannot explain the lack of influence of stage on the effect of education. The important role of comorbidity and pre-diagnosis employment (again, strongly influenced by comorbidity) indicates a challenge for the non-specialised rehabilitation of cancer patients.

The strength of this paper is its high statistical power and minimal selection bias due to use of registry data covering the whole population. The inclusion of a large healthy control sample and causal analysis of effects and interactions that this makes possible is an improvement compared to most earlier studies, which have mainly described rates of exclusion in various educational strata.

Since stage and comorbidity did not drive the inequalities, future searches for mechanisms should include a better understanding of how patients from different social strata succeed in getting a coordinated rehabilitation, including the various sectors of the Danish healthcare system involved, and of the Danish labour market’s flexibility in accepting citizens who combine disabilities with limited educational attainment. The choice of educational attainment as a measure of SEP might have affected the results. Especially in a Scandinavian country with rather high social mobility, occupational social position might better indicate the job, workplace, and labour market-related inequalities. In this case, one would expect even larger disparities in employment consequences.

## Conclusion

Based on our results, the lower employment rates among low-educated compared to highly educated female breast cancer survivors is not explained by cancer stage or comorbidity. The fact that comorbidity has little impact on the inequality may be due to a different social patterning of most comorbidity compared to breast cancer.
